# COVID-19 and Children: Reflections after Three Years

**DOI:** 10.3390/children11010010

**Published:** 2023-12-21

**Authors:** Anna Camporesi, Luigi Vetrugno, Danilo Buonsenso

**Affiliations:** 1 Pediatric Anesthesia and Intensive Care, Vittore Buzzi Children’s Hospital, 20154 Milano, Italy; 2 Department of Medical, Oral and Biotechnological Sciences, University of Chieti-Pescara, 66100 Chieti, Italy; luigi.vetrugno@unich.it; 3 Department of Woman and Child Health and Public Health, Fondazione Policlinico Universitario A. Gemelli IRCCS, 00168 Rome, Italy; danilo.buonsenso@policlinicogemelli.it; 4 Centro di Salute Globale, Università Cattolica del Sacro Cuore, 20123 Roma, Italy

Three years after the beginning of the COVID-19 pandemic, enough experience has been gained to derive reflections on the impact of SARS-CoV-2 in children.

In this editorial, we reflect not only on the overall burden of the pandemic on child health, but also on opportunities for improved responses in future similar scenarios, which predictably will arise soon or later in human history.

The whole pandemic has been definitely characterized by the false take-home message that COVID-19 was not a problem for children. This concept was mostly derived from the observation that COVID-19 was, and still is, immensely more severe in adults compared with children [1]. In fact, critical and fatal COVID-19 cases are thousands of times less frequent in children [2]. While this is a true observation, children still died during the pandemic (contribution 1). Since its discovery, SARS-CoV-2 has been the most, or one of the most (according to different periods) frequent cause of infectious disease deaths or hospitalization in children [3]. In terms of acute disease, there are several scenarios that sent children to the pediatric intensive care unit (contribution 1). COVID-19 pneumonia or ARDS such as in adults was less common than in adults, although not absent, and a new disease appeared—Multisystem Inflammatory Syndrome in Children or MIS-C—with multiple manifestations including potentially fatal cardiovascular involvement. Again, as for most diseases, COVID-19 has highlighted the enormous differences that exist between parts of the world in access to care and outcomes for pediatric patients [4,5]. Access to pediatric intensive care, for example, initially became more difficult as ICUs were re-deployed to treat adults [6,7,8].

However, severe outcomes are not limited to acute infection. SARS-CoV-2 has been characterized by a clear link with long-term disabling disease or post-acute complications, including Long COVID, MIS-C, type 1 diabetes (T1D) [9] and other rarer complications. The associations between SARS-CoV-2 and long COVID have been reported globally, and although this disease is more subtle, it is now affecting thousands of children globally, who have no access to cures or, frequently, not even access to care, being the condition still poorly characterized and often neglected in pediatric practice (REF). The possible association between SARS-CoV-2 and T1D has also been supported by important studies in major journals [9].

However, besides direct physiopathology, children have been the victims of an adult-centric system that has denied them basic rights such as education (contribution 2), potentially causing generation-long repercussions.

In addition, other scenarios indirectly harmed children. The frequent severity of acute disease in adults caused the deaths of several parents and grandparents, leaving millions of children without necessary and critical support through most of their lives. In other cases, children have suffered consequences of parental depression and mental health issues related to lockdowns, job loss, etc. (contribution 3). Lockdowns, implemented globally with few exceptions, have also caused indirect damage to children in terms of school absences, socialization, sports, mental health consequences (contribution 4-6), etc. These problems are well-accepted now and extensively discussed elsewhere (contribution 7).

Nevertheless, the experience gained during these years should serve as lessons for the future. In fact, SARS-CoV-2 has also left, at least theoretically, some lessons for the future.

The pandemic, including lockdowns, has also served as a unique “natural experiment” where SARS-CoV-2 was in essence the only circulating virus. As such, it was easy, particularly at the beginning, to notice a clear link between a hyperinflammatory disease (later called MIS-C) and a viral infection (SARS-CoV-2). This scenario has opened our understanding of frequently idiopathic conditions, such as Kawasaki disease or others, opening new research fields and potentially new preventive or therapeutic strategies. A similar consideration can be made with T1D and SARS-CoV-2, whose relationships should not come as a surprise, considering that other chronic infections (mostly from enteroviruses) are clearly linked with islet pathology, autoimmunity, and T1D onset. Again, this “natural experiment” made clear that long COVID is a chronic disabling disease associated with a recent viral infection. The interest in this disease, now affecting millions of people globally, has shed light and interest on several other post-viral diseases (mainly ME/CFS), opening new scenarios and hopes for the millions of people affected by subtle and neglected post-viral illnesses.

SARS-CoV2 has also stimulated technical advances. For example, lung ultrasound use became widely employed with a “pandemic” increase during the COVID-19 surge [10]

This increase was higher than ever before in the pediatric and adult population.

At first glance, it was thought lung ultrasound could be used to detect COVID-19-positive cases; however, it was soon evident that, in contrast with adults in children, lung ultrasound involvement was low, and the goal standard for diagnosis remained nasal or pharyngeal swabs.

Nevertheless, lung ultrasound was fundamental to recognize and predict severity in children with MIS-C or less frequent when lung involvement was present to quantify the extension of the disease and the requirement for ventilation support; as we know, it was correlated to the disease extension.

Ultimately, LUS was used to provide image modality integrated with patient clinical information to impact physician decision-making and accelerate management in COVID-19 cases.

Nevertheless, LUS was an operator-dependent exam, and the quality of images varied depending on the technique and skill of the operator, which required a steep learning curve, making challenging ultrasound studies replications and generalizable conclusions during the pandemic surge.

The need for well-trained pediatricians constitutes another significant problem in out and in-hospital teams for this technology, and the availability of expert supervisors was scarce.

In the future, developing dedicated algorithms with artificial intelligence (AI) [11] to guide LUS image acquisition through real-time feedback could help in this direction.

Maybe the AI we see today, like Siri or Alexa, will soon allow physicians to bring a probe able “to speak” about the image’s quality and acquire data in real-time, such as lung ultrasound scores, creating new opportunities that never before—or, in another way, an intelligent probe will send the image via email to a group of experts available 24/24 h to support the operator or open a deeper conversation.

Outside the use of LUS in COVID-19 infection, another critical aspect for pediatric patients will be using contrast-enhanced ultrasound (CEUS) to characterize consolidation of pneumonia vs. atelectasis, etc. helping choose treatment for reducing antibiotics use and radiation exposure in children. However, the other side of the moon is that not all that glitters during the COVID-19 pandemic will be gold today, and our feeling is that the interest in lung ultrasound has reduced, as well as the interest in course and certification.

Trying to understand how to limit SARS-CoV-2 transmission, researchers and funders have been stimulated to tackle the airborne spread of diseases. Close environments are historically linked with community respiratory infections, particularly in school children. This field of research brought us a better understanding of air as a vector of disease, exactly as other vectors such as water- and food-borne diseases, leading the development of air filters which, hopefully, may help in the future to mitigate airborne transmissions of several viruses (and not only) now causing millions of acute and long-term diseases.

Last, but not least, the pandemic can serve as an experience to improve the way we communicate about vaccinations. In terms of communications, no-vax movements have had a much stronger impact compared with pro-vax ones. Most probably, more balanced communication, based on what is currently known, what is reasonable to expect from a new vaccination campaign, the decades of experience and research behind a newly released vaccination (including a “fast-track” approach as happened with SARS-CoV-2), how all steps have been guaranteed, and properly assessed pro-risks benefits, including flexibility of strategies (based on dynamic historical periods, where priorities can change) and limiting “mandates” that are not well-seen from the public, are lessons to be taken into account for a future pandemic.

## References

[1] Zimmermann P., Curtis N. (2021). Why is COVID-19 less severe in children? A review of the proposed mechanisms underlying the age-related difference in severity of SARS-CoV-2 infections. Arch. Dis. Child..

[2] Zimmermann P., Curtis N. (2020). COVID-19 in Children, Pregnancy and Neonates: A Review of Epidemiologic and Clinical Features. Pediatr. Infect. Dis. J..

[3] Flaxman S., Whittaker C., Semenova E., Rashid T., Parks R.M., Blenkinsop A., Unwin H.J.T., Mishra S., Bhatt S., Gurdasani D. (2023). Assessment of COVID-19 as the Underlying Cause of Death among Children and Young People Aged 0 to 19 Years in the US. JAMA Netw. Open.

[4] Kitano T., Kitano M., Krueger C., Jamal H., Al Rawahi H., Lee-Krueger R., Sun R.D., Isabel S., García-Ascaso M.T., Hibino H. (2021). The differential impact of pediatric COVID-19 between high-income countries and low- and middle-income countries: A systematic review of fatality and ICU admission in children worldwide. PLoS ONE.

[5] Klazura G., Park P., Yap A., Laverde R., Bryce E., Cheung M., Bioh E., Kisa P., Kakembo N., Ugazzi M. (2023). Pediatric Surgical Waitlist in Low Middle Income Countries during the COVID-19 Pandemic. J. Surg. Res..

[6] Hill K., McCabe C., Brenner M. (2023). Impact of adapting paediatric intensive care units for adult care during the COVID-19 pandemic: A scoping review. BMJ Open.

[7] Remy K.E., Verhoef P.A., Malone J.R., Ruppe M.D., Kaselitz T.B., Lodeserto F., Hirshberg E.L.M., Slonim A.M., Dezfulian C.M. (2020). Caring for Critically Ill Adults with Coronavirus Disease 2019 in a PICU: Recommendations by Dual Trained Intensivists. Pediatr. Crit. Care Med..

[8] Fitchett E.J.A., Rubens M., Styles K., Bycroft T., Nadel S., Gómez C.M.H., Mitting R. (2022). The Pediatric Intensive Care Unit as a Critical Care Setting for Adults during the COVID-19 Pandemic: A Service Evaluation. J. Pediatr. Intensive Care.

[9] Gottesman B.L., Yu J., Tanaka C., Longhurst C.A., Kim J.J. (2022). Incidence of New-Onset Type 1 Diabetes among US Children during the COVID-19 Global Pandemic. JAMA Pediatr..

[10] Smith M.J., Hayward S.A., Innes S.M., Miller A.S.C. (2020). Point-of-care lung ultrasound in patients with COVID-19—A narrative review. Anaesthesia.

[11] Kuroda Y., Kaneko T., Yoshikawa H., Uchiyama S., Nagata Y., Matsushita Y., Hiki M., Minamino T., Takahashi K., Daida H. (2023). Artificial intelligence-based point-of-care lung ultrasound for screening COVID-19 pneumoniae: Comparison with CT scans. PLoS ONE.

